# Protective role of miR-712-3p in heatstroke-induced brain injury: involvement of neuronal lysosomal function and association with astrocytic exosome-enriched preparations

**DOI:** 10.3389/fphar.2026.1718110

**Published:** 2026-04-15

**Authors:** Yahong Chen, Jie Zhu, Shuhuan Li, Peishan Liang, Yiqiong Zhang, Guangli Ren

**Affiliations:** 1 Department of Pediatric, General Hospital of Southern Theater Command, Guangzhou, China; 2 The First School of Clinical Medicine, Southern Medical University, Guangzhou, China; 3 Department of Pediatric, The Eighth Affiliated Hospital of Southern Medical University (The First People’s Hospital of Shunde Foshan), Foshan, China; 4 Department of Pediatric, Daping Hospital, Army Medical University, Chongqing, China; 5 Guangzhou University of Chinese Medicine, Guangzhou, China

**Keywords:** astrocyte, brain injury, exosome-enriched preparations, heatstroke, miR-712-3p

## Abstract

While glia-derived exosomes have been extensively studied in heatstroke induced brain injury, the role of exosomal microRNAs (miRNAs) secreted by astrocyte remains underexplored. In this study, the viability of C8-D1A cells decreased after heat stress, apoptosis rate and the expression levels of proinflammatory cytokines such as TNF-α, IL-6, IL-1α and IL-1β increased to different degrees. The extracellular vesicles obtained by ultracentrifugation were identified *via* transmission electron microscopy (TEM), nanoparticle tracking technology Nanoparticle tracking analysis, and nanoflow cytometry (nanoFCM), which was consistent with the characterization of the exosomes. In the following sections, the collected EVs will be referred to as exosome-enriched preparations. Exosome-enriched preparations’s miRNA sequencing identified 23 differentially expressed miRNAs. Further functional analysis *via* gene ontology enrichment revealed that 46 genes regulated cell death and that 38 genes were involved in neuronal apoptosis. Kyoto encyclopedia of genes and genomes (KEGG) pathway enrichment analysis revealed that the main enriched signalling pathways were involved in biological processes such as apoptosis, inflammation, and oxidative stress. Among them, miR-712-3p was the most upregulated miRNA in the heat stress group. MiR-712-3p was overexpressed and inhibited by intranasal administration *in vivo* and cell transfection *in vitro*, and it was found that miR-712-3p could reduce brain injury and improve neuronal activity under heat stress. Moreover, RNA sequencing of neurons and transmission electron microscopy revealed that miR-712-3p can affect lysosomal function. Differential expressed genes can be involved in specific lysosome-related processes, and we predicted Atp6v1c1 associated with miR-712-3p as a candidate gene through the miRDB database. Collectively, our findings demonstrate that miR-712-3p ameliorates heat stroke-induced brain injury, with this protective effect being linked to the modulation of neuronal lysosomal function. Furthermore, the study confirms the involvement of astrocyte-derived exosome-enriched preparations in mediating this protective effect *in vitro*.

## Introduction

HS, an acute condition triggered by intense exercise or exposure to extreme heat and humidity, is characterized by a core body temperature exceeding 40 °C and neurological manifestations such as delirium, convulsions, coma, secondary multiple organ dysfunction syndrome, and systemic inflammatory syndrome (Peiris et al., 2017). With global warming, the incidence of HS is increasing annually, and the mortality of HS is also increasing significantly (Khan and Mubeen, 2025). After consulting the relevant literature, we found that brain is one of the most easily injuried organs in HS, that all HS survivors have acute neurological symptoms, and that nearly 25% have neurological sequelae (Epstein and Yanovich, 2019; Li P. et al., 2021). HS can cause brain damage through ischemia, hypoxia, inflammatory response and oxidative stress, whereas a decrease in cerebral blood flow and an increase in the metabolic rate can cause the increased permeability of the blood‒brain barrier. Inflammatory mediators can enter the central nervous system (CNS) and further aggravate brain damage, which commonly manifests as neuronal death (Yoneda et al., 2024; Wang et al., 2024). Moreover, HS-induced brain injury significantly elevates mortality and impairs patients’ quality of life, yet no effective therapeutic interventions are currently available. Therefore, there is an urgency to understand the internal mechanism of HS brain injury, and new ideas for its prevention and control are proposed.

As the predominant glial population within the central nervous system, astrocytes play multifaceted roles spanning synaptic formation, neural circuit development, and behavioral modulation. Beyond these fundamental functions, these star-shaped cells deliver essential neurotrophic support while rigorously preserving homeostasis within the cerebral microenvironment. (Giovannoni and Quintana, 2020). However, its’ role in brain injury caused by HS is unknown. Exosomes, which are extracellular vesicles, carry out cell-to-cell signalling and function by transporting highly specific substances that act on adjacent or distant cells and have been shown to contain a variety of substances (Fan et al., 2022; Isaac et al., 2021; Raghav et al., 2022; Chen et al., 2022), of which miRNAs are the most common (Isaac et al., 2021). Exosomes in HS may be involved in inflammatory and coagulation responses to mediate the damage associated with HS (Li Y. et al., 2021), such as inducing brain damage and liver damage and promoting neuronal autophagy (Li Y. et al., 2019; Li et al., 2022). We previously found that miR-466i-5p released by exosomes derived from microglia can promote neuronal apoptosis (Zhu et al., 2022), which may serve as target for the prevention of HS-induced brain injury.

Several studies have shown that astrocyte exosomes play a key role in promoting neuronal survival in neuroplasticity, immune responses, and a variety of pathological conditions (Zhang et al., 2021; Venturini et al., 2019) and involve in glioma, amyotrophic lateral sclerosis, bipolar disorder, neurodegeneration (Deng et al., 2021), ischemic brain damage (Du et al., 2021; Sun et al., 2024) and traumatic brain neurological disorders (Zhang et al., 2021; Qian et al., 2024). Brain injury mainly causes death in HS patients, but the role of astrocyte exosomes in brain injury following HS remains unclear. Therefore, we aimed to clarify the role of astrocyte-derived exosomes and their miRNAs in brain injury following HS.

## Materials and methods

### Cell culture, treatments, and CCK-8 assays

HT22 and C8-D1A cells served as hippocampal neurons and cerebellar astrocyte in mouse, respectively, were acquired from the Chinese Academy of Sciences’ Shanghai Institute of Cell Biology. The cells were cultured in DMEM/F12 media (Gibco, America) supplemented with 10% fetal bovine serum. A control group of dishes containing C8-D1A cells was cultivated for 2 h in a 37 °C incubator, while the experimental group was incubated at 42 °C for 2 h and then rewarmed at 37 °C for 0, 6, 12, or 24 h. Using the Cell Counting Kit-8 (Dojindo, Japan), cell viability was evaluated in accordance with the manufacturer’s instructions. Cells from the various treatment groups were counted and adjusted to a concentration of 1 × 105 cells/ml. Subsequently, the cell suspensions were plated at 100 μL per well in a 96-well plate. The cells were seeded in quintuplicate for each treatment group. The 96-well plate was placed in an incubator (37 °C and 5% CO2), and the cells were cultured until the appropriate time. For cell viability assay, 10 μL of CCK-8 solution was added to each well, and the cell culture plate was incubated for 2 h. The absorbance at 450 nm was measured with a microplate reader. Blank wells (culture media and CCK) and control wells (untreated cells, culture media, and CCK) were also included.

### Flow cytometry analysis of cell apoptosis via annexin V-FITC/PI staining

Detection was performed according to the manual of the Annexin V-FITC apoptosis detection kit (Invitrogen). Approximately 1 × 10^6^ cells were collected, washed with ice-cold PBS, and resuspended in binding buffer with a suitable amount of Annexin V-FITC. After 10 min incubation in the dark at room temperature, the buffer was removed by centrifugation. The cells were then resuspended in reaction buffer with propidium iodide (PI). Flow cytometry was used immediately to detect apoptosis.

### Cytokines in the astrocyte culture medium by enzyme-linked immunosorbent assay (ELISA)

Culture medium collected from the two treatment groups were directly used to determine cytokine levels. The levels of TNF-α, IL-6, IL-1α, and IL-1β were measured with a Mouse TNF-α ELISA Kit, a Mouse IL-6 ELISA Kit, a Mouse IL-1α ELISA Kit, and a Mouse IL-1β ELISA Kit (Boster, China), respectively. The standard and sample were added to a 96-well plate coated with the corresponding antibody and incubated at 37 °C for 90 min. Then, the standard and sample were aspirated, the biotin-labelled antibody was added directly to the plate, and the plate was incubated at 37 °C for 60 min. The mixture was washed three times and spun-dried, after which the avidin‒peroxidase complex was added, and the mixture was incubated for 30 min at 37 °C. The mixture was washed three times, and the mixture was spun-dried. Next, TMB developer was added to the plate and incubated at 37 °C for 15–20 min. Then, stop solution was added, the mixture was mixed well, and the absorbance at 450 nm was read with a microplate reader.

### Isolation of C8-D1A supernatant exosomes

The cell culture medium from control and heat stress C8-D1A cells were collected after rewarming for 6 h. Dead cells and cell debris were removed *via* a series of centrifugations at 4 °C (300 × g for 15 min, 2,000 × g for 20 min, and 10,000 × g for 30 min). The supernatant was subsequently filtered through a 0.22 μm cell filter and collected in a new centrifuge tube. The remaining supernatant was subjected to ultracentrifugation at 100,000 × g for 90 min at 4 °C in a Beckman Coulter Optima TM L-80XP to obtain the precipitate. Then, the precipitate was washed with 1 mL of precooled sterile PBS, which was again ultracentrifuged at 100,000 × g for 90 min at 4 °C. Finally, the precipitates were resuspended in 50 µL of sterile PBS.

### Transmission electron microscopy was used to observe exosome morphology

A total of 20 μL of the precipitate suspension was added to an electron microscope copper mesh grid for 1 min, which was subsequently stained with 2% phosphotungstic acid solution for 10 min, and filter paper was dried at room temperature. Then, the cells were visualized *via* electron microscopy (JEM-2100F, Japan). The prepared grids were examined using a transmission electron microscope operating at an accelerating voltage. Images of exosomes were captured at various magnifications using a digital camera system.

### Nanoparticle tracking analysis

Nanoparticle tracking analysis (NTA) (NS3000, Worcestershire, UK) was employed to detect the concentration and size distribution of the isolated exosomes according to the manufacturer’s instructions. Exosome samples were diluted to a concentration of 1:400 in sterile PBS, and each sample was analysed three times with NanoSight automatic analysis settings for 60 s. A comparison of the relative protein expression profiles between the control and heat stress C8-D1A-derived exosomes was performed via isobaric tags for relative and absolute quantitation technology (iTRAQ).

### Nanoflow fluorescence analysis

The biochemical characterization of the isolated exosomes was performed via a Flow NanoAnalyzer (N30E, NanoFCM, China). After the 20 μL of exosomes were diluted to 60 μL, 30 μL of the diluted exosomes were added, along with 20 μL of fluorescently tagged antibodies (CD63 and CD81, respectively). The mixture was incubated for 30 min at 37 °C in the dark. The following steps were performed twice: add 1 mL of precooled PBS, and centrifuge at 4 °C and 110,000 × g for 70 min. Ultimately, 50 μL of precooled 1 × PBS was used to prepare the sample. The performance of the NanoFCM instrument with standard products was tested first, and then, a sample test was carried out.

### Exosome labelling and *in vitro* uptake experiment

The extracted C8-D1A exosomal precipitate was resuspended in 1 × DiD dye and incubated at 37 °C for 10 min. The excess dye was washed away, and the mixture was then resuspended in DMEM/F12 for use.

HT22 cells were inoculated on cover slides at a density of 5 × 10^5^/well and cultured with complete medium until the density reached 15%–25%, after which the original medium was discarded. Next, basic medium containing labelled exosomes was added for 24 h. The tablets were subsequently fixed with 4% paraformaldehyde, permeabilized with 0.2% Triton X-100, stained with MAP2 antibody (abcam, UK), sealed with DAPI, and observed *via* fluorescence microscopy.

### Exosomal miRNA sequencing

Exosomal RNA extracted with TRIzol reagent was used to extract RNA from the exosomes. An Illumina HiSeq™ 2500 platform was used for sequencing, with a data volume of 20 M reads per sample. The total RNA or purified sRNA fragment of the sample was extracted. The 3′ and 5′ end junctions were ligated, reverse transcribed into cDNA, and then amplified *via* PCR. The target fragment library was then recovered *via* gum cutting, and the libraries passed quality control. Libraries were sequenced on the machine. Joints at the ends of reads and reads with fragment lengths <17 nt and low-quality reads were removed to complete the initial filtering of data and obtain high-quality data. The clean reads were compared with the reference genome to obtain a genome-wide read distribution map, and the clean reads were annotated with ncRNA classification. The identified miRNAs were subjected to expression calculations, miRNA expression clustering, and differentially expressed miRNA analysis between samples. For miRNAs with significant differences, the target genes of the miRNAs were further predicted, and GO and KEGG biological pathway enrichment analyses were performed for the target genes. We used Bowtie to compare the clean reads from the sequencing data to those from the reference genome (RIBOBIO).

### Sequencing data analysis

All the quantitative data are expressed as the means ± standard deviations; a t-test was used for comparisons between two groups, and one-way analysis of variance (ANOVA) was used for comparisons of multiple groups. miRNA candidates with >2-fold up- or downregulation and p<0.05 were considered significantly altered miRNAs. Target miRNAs were predicted as possible target molecules by TargetScan, and the predicted genes were then subjected to GO analysis, functional annotation analysis, and KEGG database analysis to determine the functional pathways involved. The 2−DDCt method was used to explore the expression of target genes.

### Real-time quantitative PCR

Total RNA was extracted *via* TRIzol lysis (Invitrogen, CA, USA), and the concentration and quality of the RNA were measured. The reaction system was configured with a Takara TB Green Premix Ex Taq kit, and a QuantStudio 7 Flex real-time fluorescence quantitative PCR system was used for quantitative PCR to detect the expression levels of target genes. Primers were shown as follows: miR-712-3p, F, 5′-TGC​GAG​TCA​CCC​CCG​G-3′, R, 5′-GTC​GTA​TCC​AGT​GCA​GGG​TCC​GAG​GTA​TTC​GCA​CTG​GAT​ACG​ACC​AAC​AC-3’; miR-3095-3p, F, 5′-CGC​GTG​GAC​ACT​GGA​GAG​AGA-3′, R, 5′-GTC​GTA​TCC​AGT​GCA​GGG​TCC​GAG​GTA​TTC​GCA​CTG​GAT​ACG​ACA​AAA​GC-3’; miR-712-5p, F, 5′-GCT​CCT​TCA​CCC​GGG​C-3′, R, 5′-GTC​GTA​TCC​AGT​GCA​GGG​TCC​GAG​GTA​TTC​GCA​CTG​GAT​ACG​ACG​GTA​CC-3’; miR-181b-5p, F, 5′-CGA​ACA​TTC​ATT​GCT​GTC​GG-3′, R, 5′-GTC​GTA​TCC​AGT​GCA​GGG​TCC​GAG​GTA​TTC​GCA​CTG​GAT​ACG​ACA​ACC​CA-3’; mir16-5P, F, 5′-CGC​GTA​GCA​GCA​CGT​AAA​TA-3′, R, 5′-GTC​GTA​TCC​AGT​GCA​GGG​TCC​GAG​GTA​TTC​GCA​CTG​GAT​ACG​ACC​GCC​AA-3’; miR-1947-5p, F, 5′-GCG​AGG​ACG​AGC​TAG​CTG​A-3′, R, 5′-GTC​GTA​TCC​AGT​GCA​GGG​TCC​GAG​GTA​TTC​GCA​CTG​GAT​ACG​ACC​AGC​AC-3’; U6, F, 5′-CTC​GCT​TCG​GCA​GCA​CA-3′, R, 5′-AAC​GCT​TCA​CGA​ATT​TGC​GT-3’; Reverse primer: 5′-AGT​GCA​GGG​TCC​GAG​GTA​TT-3’.

### miR-712-3p mimic transfection and CCK-8 assays

The miR-712-3p mimics and miR-712-3p inhibitor were purchased from RiboBio. To transfect HT22 cells, equal volumes of miR-712-3p mimics or inhibitors were mixed with transfection reagent (Zeta Life, America), and the mixture was incubated for 15 min at room temperature (RT). For six-well plate transfections, the miR mixture was mixed with 5% FBS medium (DMEM/F12) with no penicillin‒streptomycin and added to each well of the HT22 plate for 24 h. The medium was then changed to 10% FBS medium without penicillin‒streptomycin. The control group was treated as a blank, 5% FBS medium without penicillin‒streptomycin was added simultaneously to the heat stress group during transfection, and the medium was changed simultaneously to 10% FBS medium without penicillin‒streptomycin 24 h later.

Control group cells were maintained at 37 °C with 5% CO2, whereas heat stress group cells were subjected to 42 °C for 2 h followed by rewarming at 37 °C under the same CO2 conditions. After rewatering for 6 h, cell viability was assessed with a Cell Counting Kit-8 (Dojindo, Japan) according to the manufacturer’s instructions.

### RNA sequencing and data analysis

RNA was extracted from cells with TRIzol reagent. A NanoDrop ND-2000 was used to determine the A260/A280 absorbance ratio of the RNA samples, and an Agilent Bioanalyzer 4150 was used to determine the RIN value of the RNA. PE libraries were prepared according to the ABclonal mRNA-seq Lib Prep Kit. mRNA was purified from 1 μg of total RNA *via* oligo (DT) magnetic beads, and then, mRNA fragmentation was performed in ABclonal First Strand Synthesis Reaction Buffer. The first strand of cDNA was subsequently synthesized *via* random primers and reverse transcriptase (RNase H), and the second strand of cDNA was subsequently synthesized *via* DNA polymerase I, RNAseH, buffer, and dNTPs. The synthesized double-stranded cDNA fragment was ligated with the linker sequence for PCR amplification. The PCR products were purified, and library quality was assessed *via* an Agilent Bioanalyzer 4150. Sequencing was performed with the Illumina NovaSeq 6000 sequencing platform. The FASTQ-formatted raw data were first processed *via* Perl scripts to remove the linker sequence. Low-quality reads (low-quality reads with base mass values less than or equal to 25 accounting for more than 60% of the total reads) and N (n representing undeterminable base information) accounting for more than 5% of the total reads were filtered out, and clean reads were obtained for subsequent analysis. The clean reads were compared with the reference genome *via* HISAT2 software (http://daehwankimlab.github.io/HISAT2/) to obtain mapped reads for subsequent analysis. Differential expression analysis of genes between groups was performed *via* Deseq2 (http://bioconductor.org/packages/release/bioc/html/DESeq2.html), with default screening thresholds for differentially expressed genes as follows: | log2FC | > 1 and Padj <0.05. Finally, with the KEGG pathway as the unit and the reference genome as the background, Fisher’s exact test was used to analyse and calculate the significance level of gene enrichment in each pathway to identify the metabolic and signal transduction pathways that were significantly affected.

### The morphology of lysosomes was observed *via* TEM

The cells from each group were collected, centrifuged (1000 rpm, 5 min) and washed twice with PBS, followed by the slow addition of 200 μL of 3% glutaraldehyde at 4 °C to the medulla oblongata wall overnight fixation, followed sequentially by rinsing with 0.1 M PBS for 2 h and fixing with 1% osmium tetroxide for 100 min. The fixed samples were then rinsed with 0.1 M PBS for 5 min. The fixed samples were immersed in 50% ethanol, 70% ethanol, 80% acetone I, 80% acetone II, 90% acetone I, 90% acetone II, 100% acetone I, and 100% acetone II for 10 min to dehydrate. The dehydrated samples were soaked in a 1:1 mixture of 100% acetone and embedding agent for 1 h and then placed in a 37 °C incubator overnight. Then, the pure embedding agent was dropped on the bottom of the capsule, and the soaked cells were transferred into the capsule and filled with the embedding agent. Finally, the sections were observed and photographed under a biometric projection electron microscope.

### Animals

Male C57BL/6 mice (weight, 20–30 g; age, 6–8 weeks; Ruige Biological Technology, Guangzhou, China) were maintained under controlled environmental conditions (12 h light/dark cycle, humidity, 55% ± 5%, temperature, 25 °C ± 1 °C) at the Experimental Animal Center of the General Hospital of Southern Theatre Command of the PLA and were given free access to standard laboratory chow and water. After the experimental model was established, the mice were anesthetized by intraperitoneal injection of 1% pentobarbital sodium at a concentration of 40–45 mg/kg of body weight, and then sacrificed by cervical dislocation. The animal ethics committee of the General Hospital of Southern Theatre Command of the PLA approved this study.

### Heat stress mouse model and cooling treatment

The mice in the control group were maintained at 25 °C ± 0.5 °C and a humidity of 35% ± 5%. The mice in the heat stress group were placed in a prewarmed incubator at 37 °C ± 0.5 °C and a relative humidity of 70% ± 5% in the absence of food and water. The rectal core temperature (Tc) was continuously monitored with a rectal thermometer. The mice were removed from the incubator and allowed to cool at an ambient temperature of 25 °C ± 0.5 °C when the Tc reached 42 °C, or the mice were lethargic, stowed without arching back, and exhibited no avoidance behavior when exposed to stimulation.

### Grouping and experimental procedures

The effects of the miR-712-3p agomir (RiboBio, Guangzhou, China) and miR-712-3p antagomir (RiboBio, Guangzhou, China) on neuronal apoptosis were explored. Twenty nine mice were randomly divided into a blank control group (n = 5), NC agomir group (n = 6), NC antagomir group (n = 6), miR-712-3p agomir group (n = 6), and miR-712-3p antagomir group (n = 6). In the NC agomir group, NC antagomir group, miR-712-3p agomir group and miR-712-3p antagomir group, the mice were given the NC agomir, NC antagomir, miR-712-3p agomir or miR-712-3p antagomir with 24 µL (1 nmol, 40 nmol/mL) by nasal delivery, and blank control mice were given an equal volume of physiological saline and 1 µL per drop, altering drops between the left and right nose, finishing within 1 min. The mice in each group were subjected to heat stress 24 h after nasal delivery and then sacrificed 24 h after heat stress, and the relative expression of miR-712-3p was verified *via* quantitative real-time PCR (qRT‒PCR).

### HE assay

The wax blocks cooled at −20 °C were placed in a paraffin microtome (thickness of 4 μm), the slices were placed in warm water at 40 °C in a sheet spreading machine to flatten the tissues, and the tissues were picked up on slides and baked in an oven at 60 °C for 2 h.

The paraffin-embedded sections were soaked successively in xylene I, xylene II, anhydrous ethanol I, and anhydrous ethanol II for 20 min, 20 min, 5 min, and 5 min, after which they were washed with anhydrous ethanol for 20 s, after which the ethanol in the sections was rinsed with tap water. The dewaxed sections were placed in hematoxylin for 3–5 min, washed until colourless, placed in differentiation solution for 3–5 s, quickly washed, placed in anti-blue solution for 3–5 s and quickly washed. The samples were then soaked in 85% ethanol, 95% ethanol, eosin dyeing solution, anhydrous ethanol I, anhydrous ethanol II, anhydrous ethanol III, n-butanol, xylene I, and xylene II in each cylinder for 3–5 min. The slices were removed, air dried, sealed with neutral gum, and observed with a standing optical microscope.

### Nissl’s staining

Paraffin-embedded C57 mouse brain tissue sections were dewaxed in water, stained with Nysk’s dye, dried, treated with xylene, and finally sealed with neutral gum, and the changes in Nysk bodies were observed under a standing microscope.

### Statistical analysis

GraphPad 8.0 (La Jolla, CA) software was used to analyse the data, and those with metrological data that were normally distributed were described statistically as the means ± SDs (those that were not normally distributed were described as medians and interquartile ranges). Counting data are described statistically in terms of relative numbers. One-way analysis of variance (LSD-T method was used to compare two groups). Two-way analysis of variance was used for two-factor analysis. There was a significant difference at P < 0.05 (two-sided). All the experiments were repeated 3 times independently.

## Results

### HS induced astrocyte apoptosis and the release of inflammatory factors

**FIGURE 1:**
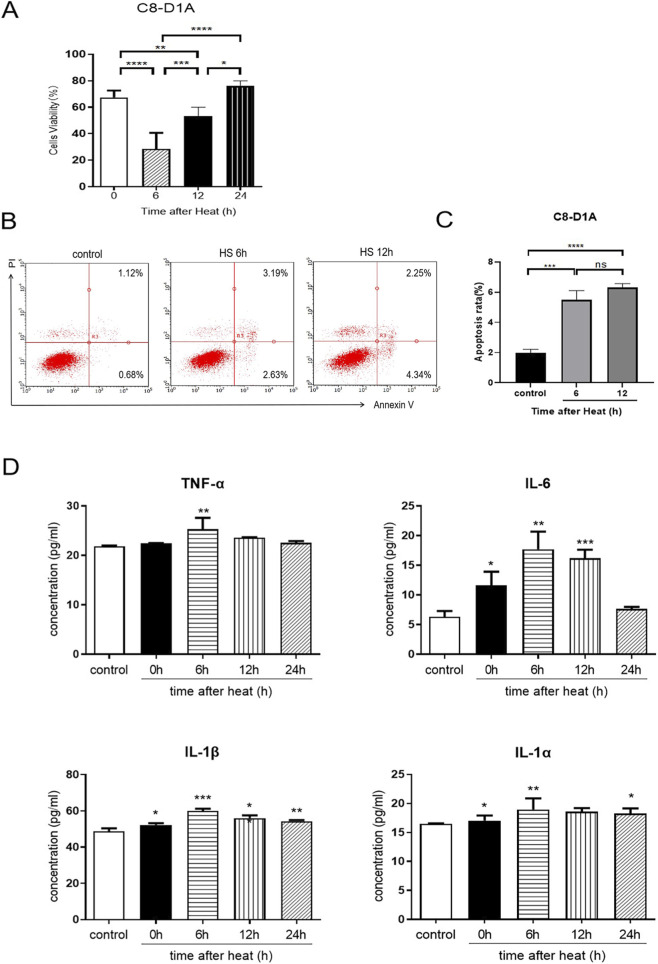
Changes in the viability and function of C8-D1A cells after HS. **(A)** Comparison of cell viability at 0 h, 6 h, 12 h and 24 h after HS. The cell viability decreased most obviously in the 6 h group. **(B)** Apoptosis of C8-D1A was detected by flow cytometry at 6 h and 12 h after HS. **(C)** Comparison of C8-D1A cells at 6 h and 12 h apoptosis rate after HS. **(D)** Levels of inflammatory factors in the culture supernatant of C8-D1A cells after different rewarming durations after HS. *P < 0.05, **P < 0.01, ***P < 0.001, ****P < 0.0001.

After heat stress, the viability of C8-D1A cells decreased significantly after 6 h and 12 h of rewarming (p < 0.05), and the viability of C8-D1A cells in the rewarming 6 h group decreased more significantly than that in the rewarming 12 h group. C8-D1A cells rewarmed for 24 h exhibited significantly higher viability compared to those rewarmed for 12 h (p < 0.05), with no significant difference from the 0 h rewarming group, suggesting recovery of cell viability by 24 h (Figure 1A). C8-D1A cell apoptosis rate was significantly greater in the heat stress group than in the control group at 6 h and 12 h of rewarming (p < 0.05) referred to Figures 1B,C.

The culture medium of C8-D1A cells were collected at 0, 6, 12 and 24 h after rewarming, and the ELISA results revealed that the expression levels of the proinflammatory cytokines TNF-α, IL-6, IL-1α and IL-1β were increased to varying degrees (Figure 1D). The level of TNF-α increased significantly after 6 h and recovered after 24 h of rewarming. The level of IL-6 increased significantly at 0 h, 6 h, and 12 h after rewarming, peaked at 6 h, and recovered at 24 h. Compared with those in the control group, the levels of IL-1β were significantly increased at 0, 6, 12 and 24 h after rewarming. And the levels of IL-1α were significantly increased at 0, 6 and 24 h after rewarming. In conclusion, heat stress can promote the release of proinflammatory cytokines by astrocytes.

### Astrocyte-derived exosome-enriched preparations after HS affect neuronal viability

**FIGURE 2:**
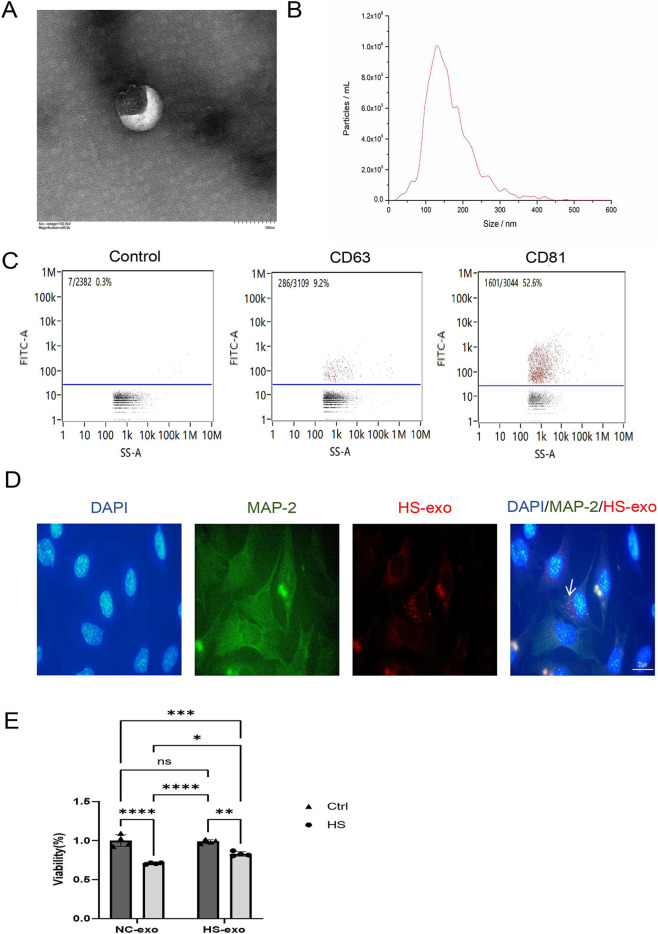
Identification of exosomes in C8-D1A cells after HS and their effects on neuronal function. **(A)** The morphology of exosomes in the culture supernatant of C8-D1A cells after HS was observed via TEM. The size of the bilayer vesicle structure is approximately 100 nm. Scale bar: 100 nm. **(B)** NTA was used to measure the size distribution of astrocyte exosomes after HS. The size of the vesicles was concentrated at 150 nm. **(C)** Levels of representative exosome surface markers. **(D)** Neurons uptake astrocyte-derived exosome. The white arrows indicate the uptake of exosomes. Scale bar: 20 μm. **(E)** Astrocyte-derived exosomes increase neuron viability. *P < 0.05, **P < 0.01, ***P < 0.001, ****P < 0.0001.

The extracellular vesicles in the supernatant of heat-stressed C8-D1A astrocytes were isolated *via* ultracentrifugation. TEM revealed that the vesicles were typical double-layer membrane-like structures with intact envelopes, which are substances with low electron density (Figure 2A). NTA detection of the isolated extracellular vesicles revealed that the size of these vesicles was concentrated at 150 nm (Figure 2B). In addition, we examined expression of the exosome marker CD63 and CD81 in extracellular vesicles of heat stress C8-D1A culture supernatants *via* nanoFCM (Figure 2C). The results indicated that the C8-D1A extracellular vesicles extracted by ultracentrifugation were exosome-enriched preparations.

To clarify the effect of astrocyte-derived exosomes on neuronal function, we first labelled the exosomes *via* DiD dye and then cocultured them with HT22 cells. HT22 was found to take up C8-D1A-derived exosomes (Figure 2D), suggesting that exosome-enriched preparations from astrocytes may affect neuronal function. We then further examined the viability of HT22 cells subjected to heat stress after 24 h of coculture with C8-D1A exosome-enriched preparations (Figure 2E) and found that, compared with that in the control group, the viability of HT22 cells in the heat stress group was significantly decreased after the uptake of control group C8-D1A exosomes, whereas that of HT22 cells in the heat stress group was significantly increased after the uptake of heat stress-derived C8-D1A exosomes, suggesting that exosome-enriched preparations from heatstroke astrocytes specifically alleviates brain injury in heatstroke. This activity is closely associated with a vesicle fraction possessing the characteristic size and marker profile of exosomes, strongly implicating exosomes as key mediators in this process.

### The expression of miR-712-3p in C8-D1A-derived exosome-enriched preparations was upregulated after HS

**FIGURE 3:**
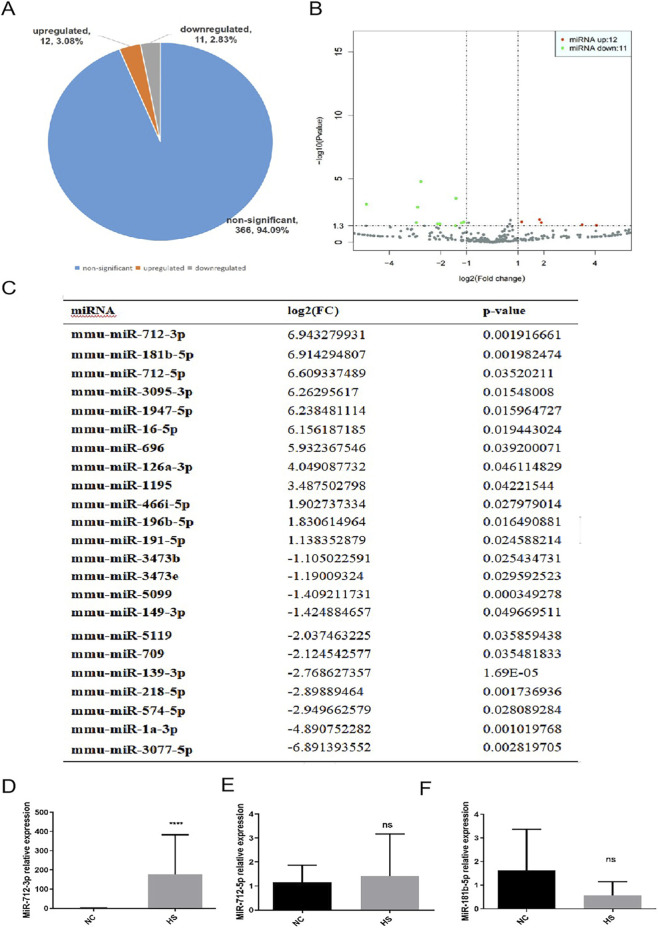
Heat stress altered the miRNA expression pattern of C8-D1A-derived exosomes. **(A)** Proportion of differentially expressed genes (upregulated, downregulated and nonsignificant). Among them, 12 miRNAs were upregulated, and 11 miRNAs were downregulated. **(B)** Volcano plots for differential expression analysis of miRNAs among samples. **(A)** Horizontal axis: Fold change in miRNA expression in different samples; Vertical axis: Statistically significant difference in miRNA expression; Red dots: Significantly upregulated miRNAs; Green dots: Significantly downregulated miRNAs. **(C)** Differentially expressed miRNAs in C8-D1A-derived exosomes after heat stress. **(D–F)** Expression of C8-D1A cell-derived exosomal miRNAs after heat stress. *P < 0.05, **P < 0.01, ***P < 0.001, ****P < 0.0001 vs. the NC group.

The supernatants of C8-D1A were collected from the control group and rewarmed for 6 h after heat stress. The exosome-enriched preparations were isolated *via* ultracentrifugation and subjected to high-throughput sequencing to compare miRNA expression. Compared with those of the control, 366 miRNAs (94.09%) did not change significantly after heat stress, 12 miRNAs (3.08%) were upregulated, and 11 miRNAs (2.83%) were downregulated (Figure 3A). The different miRNA expression in exosomes between heat stress and control group is shown in Figure 3B. The 23 differentially expressed miRNAs are detailed in Figure 3C, with miR-712-3p being upregulated most significantly. We then performed hierarchical clustering analysis of these 23 differentially expressed miRNAs.

**FIGURE 4:**
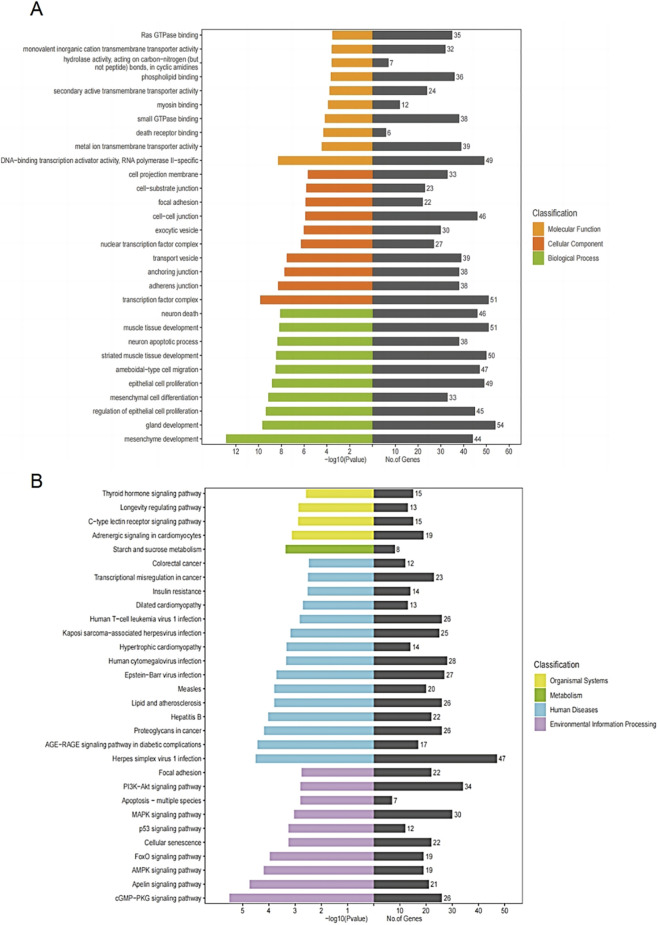
GO functional and KEGG enrichment classification map of miRNA candidate target genes. **(A)** GO functional enrichment classification map. **(B)** KEGG pathway enrichment classification map.

To verify that miR-712-3p is the miRNA with the most obvious upregulation in astrocyte exosome-enriched preparations after heat stress, we verified its expression level by qPCR and found that its expression increased most significantly (Figures 3D–F). To determine specific biological effects, we used GO analysis. As shown in Figure 4A, 23 miRNAs were significantly altered in the heat stress group and found that 46 genes regulated cell death and 38 genes were involved in the neuronal apoptosis. Therefore, we speculate that astrocytes may be involved in brain injury after HS through exosomes.

Moreover, we performed KEGG pathway enrichment analysis on these 23 miRNAs and revealed that the PI3K-AKT, MAPK, p53, FoxO pathway, AMPK and cGMP-PKG pathway were the major enriched signalling pathways, which were involved in biological processes such as apoptosis, inflammation and oxidative stress (Figure 4B). Among them, 19 genes were enriched in the AMPK signaling pathway, including: Akt1, Ccna2, Ccnd1, Creb1, Creb5, Igf1, Igf1r, Insr, Irs1, Map3k7, Pfkfb3, Pfkl, Pik3r2, Prkag3, Rab11b, Rheb, Scd3, Scd4, and Slc2a4. Additionally, 26 genes were enriched in the cGMP-PKG signaling pathway, such as: Adra1b, Akt1, Atp1a2, Atp2a2, Atp2b3, Calm1, Calm2, Creb1, Creb5, Gata4, Gna13, Insr, Irs1, Mef2a, Myh6, Mylk4, Nfatc1, Nfatc4, Nppa, Pik3r5, Plcb1, Ppp1cb, Prkce, Slc8a1, Slc8a3, and Srf. These results suggested that exosome-enriched preparations from astrocytes may activate these signalling pathways to induce or inhibit brain injury.

### miR-712-3p alleviated HS-induced brain injury

**FIGURE 5:**
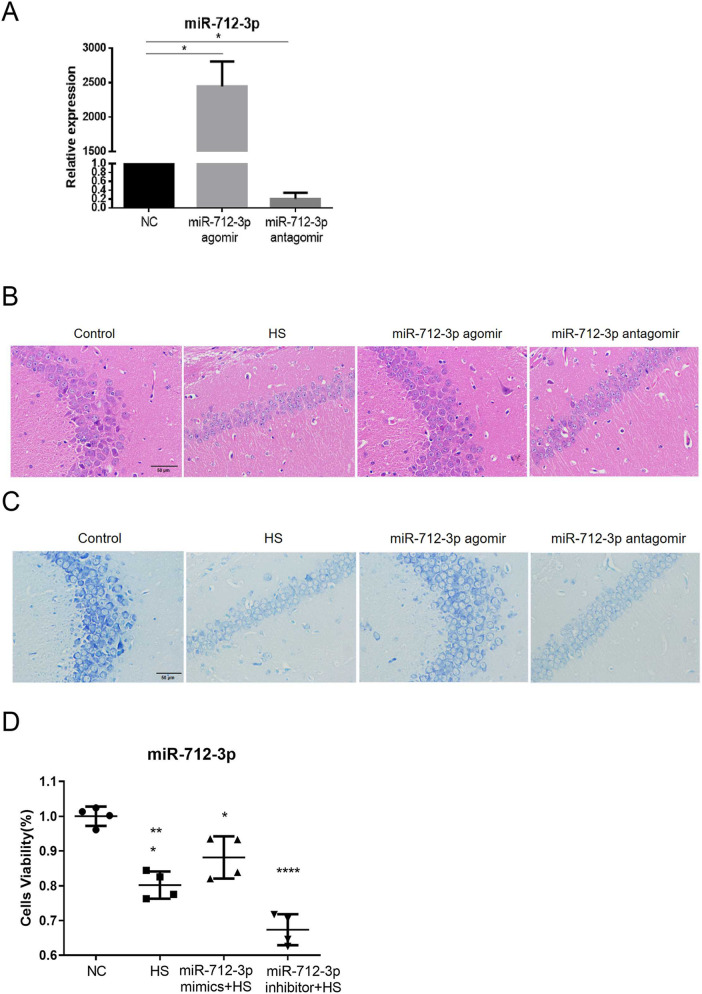
MiR-712-3p protected brain function during heat stress. **(A)** The expression level of miR-712-3p in brain tissue. **(B)** HE staining of mouse brain tissue. **(C)** Nissl staining of mouse brain tissue. Scale bar: 50 μm. **(D)** miR-712-3p protected neuronal function during heat stress. *P < 0.05, **P < 0.01, ***P < 0.001, ****P < 0.0001 vs. the NC group. ^#^ *P* < 0.05, ^##^ *P* < 0.01, ^###^ *P* < 0.001, ^###^ *P* < 0.0001 vs. the HS group.

Given the NC agomir, NC antagomir, miR-712-3p agomir or miR-712-3p antagomir in each experimental group by nasal delivery, and blank control mice were given an equal volume of physiological saline. The brain tissues of C57 mice after intranasal administration were ground, and RNA was extracted. The expression levels of miR-712-3p were verified *via* real-time quantitative PCR (Figure 5A), and there was no significant difference in the expression of miR-712-3p compared with that in the control group. The expression of miR-712-3p in the overexpression group was significantly increased (p < 0.05), whereas the expression of miR-712-3p in the inhibitor group was significantly decreased (p < 0.05), indicating that the drug could enter the brain *via* the nose.

To further clarify the effect of miR-712-3p on neural function, we performed HE and Nissl staining on the brain tissue of each group of C57BL/6J mice (Figures 5B,C) and found that, compared with those of the control mice, the number of Nissl bodies was significantly greater in the mice overexpressing miR-712-3p. However, vacuolar degeneration and the number of Nissl bodies were significantly decreased in mice with inhibited miR-712-3p expression. In addition, we constructed HS cell model after transfection of miR-712-3p mimics and inhibitor into HT22 and then examined cell viability with CCK-8 reagent and found that cell viability was significantly increased in the miR-712-3p mimics group and the decrease of cell viability in the miR-712-3p inhibitor group (Figure 5D).

The administration of miR-712-3p *via* the intranasal route attenuated brain injury in the heatstroke model. Mechanistically, this effect aligns with our *in vitro* evidence demonstrating the miRNA’s capacity to modulate astrocyte reactivity. We therefore propose that the therapeutic benefits observed *in vivo* may, at least in part, be mediated through the functional regulation of astrocytes. Therefore,we hypothesize that miR-712-3p can protect neuronal function under HS.

### MiR-712-3p may affect lysosomal function to protect neurons under HS

**FIGURE 6:**
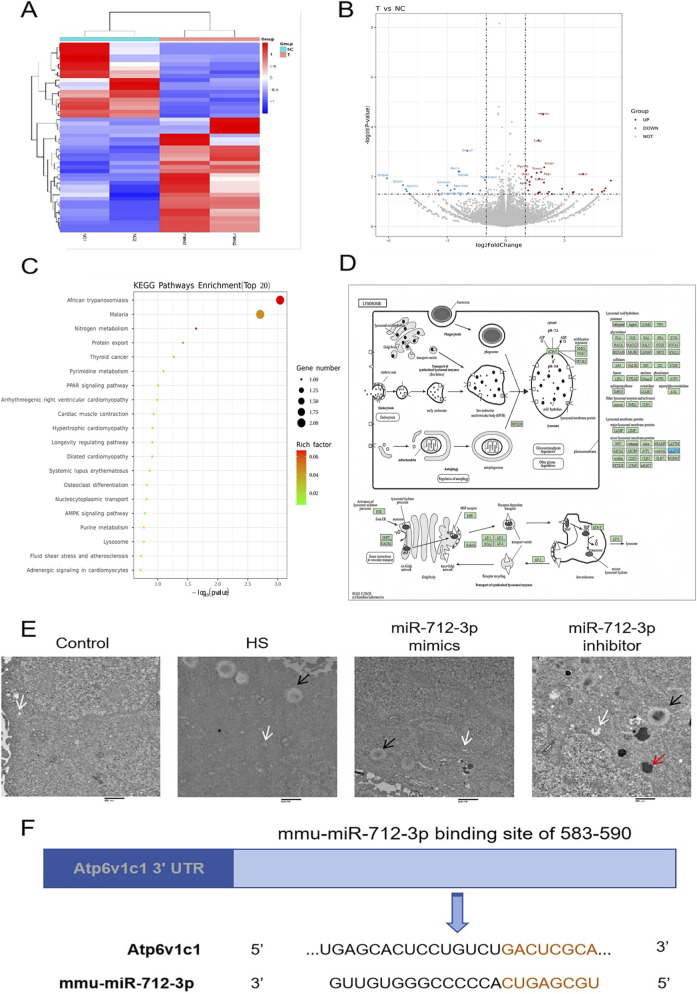
MiR-712-3p altered the transcriptome expression pattern of HT22 cells. **(A)** Heatmap of differentially expressed mRNAs. **(B)** Volcano plot of differentially expressed mRNAs. **(C)** KEGG pathway enrichment classification map of mRNA candidate target genes. **(D)** DEG pathway of lysosome. **(E)** MiR-712-3p affects the number and morphology of HT22 lysosomes and lipid droplet formation. **(F)** The binding sites of mmu-miR-712-3p in the Atp6v1c1 3′ transcription region predicted by TargetScan. White arrow: lysosome; black arrow: lipid droplet; red arrow: lipofuscin. Scale bar: 800 nm.

We subsequently collected HT22 cells overexpressing miR-712-3p for RNA sequencing to explore the signalling pathways through which miR-712-3p affects neurons and found that 48 mRNAs were differentially expressed; of these, 29 mRNAs were upregulated, and 19 mRNAs were downregulated (Figure 6A), with the top 10 mRNAs whose magnitude of change is already indicated in Figure 6B.

To further explore the signalling pathways through which miR-712-3p affects neurons, we performed KEGG pathway enrichment analysis on the above 48 differentially expressed mRNAs, and miR-712-3p was found to affect neuronal function possibly through lysosomes (Figure 6C). The specific lysosomal-related processes that the DEGs were involved in were illustrated in Figure 6D.

Therefore, we utilized transmission electron microscopy to observe HT22 lysosome after transfection of miR-712-3p mimics and inhibitor (Figure 6E) and found that few lysosomes were observed in control HT22 cells. In the following text, HT22 cells subjected to heat stress are referred to as the “heat stress group”. HT22 cells transfected with the miR-712-3p mimic and subjected to heat stress are hereinafter referred to as the “miR-712-3p mimic group”, whereas those transfected with the miR-712-3p inhibitor prior to heat stress are designated as the “miR-712-3p inhibitor group”. The number of autophagosomes and lipid drops in HT22 cells induced by heat stress was significantly greater than that in control HT22 cells. HT22 cells in the miR-712-3p mimic group exhibited significantly fewer autophagosomes and lipid drops than those in the heat stress group, with isolated lipofuscin granules observable in a small number of cells. In contrast, miR-712-3p inhibitor group led to a marked increase in lipofuscin granules. This morphological rescue is consistent with a normalization of the autophagic-lysosomal pathway and suggests a potential amelioration of lysosomal dysfunction or autophagic flux. Through miRDB and GeneCards, we predicted that miR-712-3p might affect lysosomal function by binding to Atp6v1c1, and the specific binding sites were shown in Figure 6F.

## Discussion

Astrocytes, crucial cells of the CNS, dynamically change the condition of the brain and mediate the inflammatory response to infection and brain injury (NJ and DA, 2018). In accordance with the literature, HS-treated mice experienced a hypothermic period of 500–700 min after rewarming. During this time, the shift in inflammatory factors was most noticeable, and it gradually subsided after 6 h (Li H. et al., 2019; Klett et al., 2015). The lack of a consistent hypothermia time point, however, might be due to variations in modelling and rewarming settings as well as animal age and species (Biedenkapp and Leon, 2013). It is hypothesized that heat stress may cause some damage to astrocytes, which secrete proinflammatory factors to participate in neuroinflammatory reactions, especially after rewarming for 6 h. In this study, we found that astrocyte viability decreased and apoptosis increased after heat stress and that the levels of TNF-α, IL-6, IL-1α, and IL-1β secreted by the cells increased significantly after rewarming for 6 h after heat stress. The intracellular heat shock protein and chaperone families may play a part in the reduction in inflammatory factors 24 h after rewarming. In addition to the increase in anti-inflammatory factors and decrease in proinflammatory factors, the inflammatory response is balanced, and cell viability is restored (Epstein and Yanovich, 2019). Since the cells themselves may also undergo apoptosis during the process of proliferation, which may be accelerated under conditions of poor cell viability, the increase in apoptosis was more noticeable when the cells were rewarmed for 12 h. We still believe that rewarming for approximately 6 h represents an acute response phase, which is in line with the team’s earlier findings (Gao et al., 2021; Zou et al., 2018), even if the two experimental approaches have distinct underlying concepts.

These findings are in line with the observed increase in astrocyte reactivity as well as the severe morphological, molecular, and functional alterations that occur throughout the disease state. In addition to affecting neuronal function, promoting the capacity of exosomes and aggravating neuroinflammation, activated astrocytes can release a range of active substances, including chemokines, neurotoxic substances, and inflammatory mediators, therefore, the activated astrocytes may appear death soon. They also change the capacity of astrocytes to maintain brain homeostasis and are essential for the survival and recovery of other cells in the CNS (You et al., 2020; Han et al., 2021). Thus, we hypothesised that heat stress would encourage astrocytes to release exosome and impact neuronal function, but this hypothesis requires additional experimental confirmation.

Extracellular vesicles released by astrocytes under normal circumstances have been found to have neurotrophic and neuroprotective qualities. Extracellular vesicles generated by astrocytes can transport a variety of substances involved in neuronal survival to shield neurons from neurotransmitter toxicity and promote synaptic development in the absence of nutrition (Upadhya et al., 2020). Exosomes are 30–150 nm in diameter and belong to extracellular vesicles. Astrocyte exosomes decrease the degree of neuronal apoptosis induced by hypoxia-ischemia (Du et al., 2021). Neuronal activity may be impacted by modifications in the intracellular pH balance, glutamate clearance, Ca^2+^ signalling, or the glycolytic rate (Dallérac et al., 2018). While we acknowledge that the absence of a vesicle disruption control limits definitive attribution of the observed effects to intact exosomes alone, the exosomal component remains a critical biological entity within the preparation, and its role as a primary mediator is strongly implicated by the collective evidence. To conclusively verify the functional necessity of vesicle integrity and establish a definitive causal link, future investigations should incorporate rigorous methodologies, including vesicle disruption assays, immunoisolation techniques targeting specific surface markers, and genetic perturbation of key exosome biogenesis or secretion pathways.

MicroRNAs (miRNAs) are small noncoding RNA molecules that range in size from 18 to 23 NTs. Through posttranscriptional regulation of genes, they play a role in cell proliferation, differentiation, and death. They also regulate nearly all the cellular processes that have been examined to date (Krol et al., 2010). Exosome-derived miRNAs have been the subject of research since it was discovered that neuroglia miRNAs, including astrocyte and microglia exosomes, play significant roles in the development of brain injury (Liu et al., 2023; He et al., 2023). Several studies have demonstrated that miRNAs are involved in traumatic brain injury, intracerebral hemorrhage, stroke, hypoxic-ischemic brain injury, epilepsy, and other diseases of the CNS (Zhu et al., 2023; Kashif et al., 2021; Kadir et al., 2022; Peeples, 2023; Morris et al., 2021; Pir et al., 2024), and the same is true for miRNAs in extracellular vesicles (Toor et al., 2025).

Among these, astrocyte exosomal miR-148a-3p inhibits neuroinflammation and restores nerve function in traumatic brain injury by modulating the microglial phenotype (Qian et al., 2024), whereas exosomal miR-873a-5p can reduce ischemic neuroinflammation by regulating NLRP3-mediated pyrodeath (Sun et al., 2024). We employed high-throughput sequencing to identify the differential expression of miRNAs in astrocytic exosomes under heat stress to better investigate the role of astrocytic exosomes in heatstroke-induced brain injury. These findings demonstrated that the kinds of astrocyte exosome miRNAs were altered in response to heat stress. Additional GO functional enrichment analysis revealed that 38 genes were involved in the neuronal apoptosis process and that 46 genes controlled cell death. KEGG pathway enrichment analysis revealed the primary signalling pathways involved in oxidative stress, inflammation, apoptosis, and other biological processes. Through signalling pathways such as the PI3K-Akt, MAPK, p53, FOXO, AMPK, and cGMP-PKG pathways, we hypothesized that these differentially expressed miRNAs might control their own and neuronal apoptosis while also inducing oxidative stress and an inflammatory response that further damages cells. In our study, miR-712-3p was the most obvious miRNA upregulated during heat stress. Therefore, we hypothesized that miR-712-3p might affect the function of neurons through the abovementioned process. However, as one of the miRNA families, miRNAs have not been reported thus far; thus, studying the effects of miRNAs on neurons under HS is highly valuable. In this study, we constructed a C57 heat shock mouse model and an HT22 heat stress cell model to compare the state of neurons after the miR-712-3p expression level was increased or inhibited. Moreover, the apoptosis and cell viability of neurons overexpressing miR-712-3p were decreased, whereas those inhibiting miR-712-3p were further decreased. The sufficiency of miR-712-3p overexpression to recapitulate the neuroprotective effects of the complete exosome preparation provides direct evidence for its role as an essential contributor to the observed therapeutic action. These results suggest that miR-712-3p may exert a protective role in brain injury from HS by modulating lysosomal function. Furthermore, astrocyte-derived exosome-enriched preparations have been shown to alleviate HS-induced brain injury *in vitro*. Definitive attribution of the observed effects to exosomal miR-712-3p requires further validation through the use of critical controls. Specifically, it will be essential to directly compare the functional outcomes of exosomes from miR-712-3p–inhibited astrocytes and EV-depleted conditioned medium against those of unmodified astrocyte-derived exosomes. We identified 49 mRNAs differentially expressed by RNA sequencing and performed KEGG pathway enrichment analysis on these 49 mRNAs and revealed that lysosomes may be involved in the process by which miR-712-3p protects against HS-induced brain injury.

Yang Xuesen’s team previously discovered that heat stress can cause lysosomal dysfunction that results in brain damage, which can manifest as increased autophagosomes and decreased autophagic flux after heat stress, suggesting that overactivation of autophagy may further promote neuronal death (Yi et al., 2017). Lipofuscin is widely recognized as a hallmark of aging and oxidative stress. Its accumulation has been shown to disrupt autophagic-lysosomal trafficking. Under conditions such as lysosomal alkalinization or increased oxidative stress, lipofuscin further impairs autophagic flux, establishing a feedback loop that perpetuates degradative dysfunction. While neurons with a high lipofuscin load may remain relatively stable under basal conditions, neuronal death can lead to the release of intracellular lipofuscin into the extracellular milieu, potentially aggravating oxidative stress in surrounding neurons (Dougnon and Matsui, 2025). This is also consistent with our observations under electron microscopy. We hypothesize that heat stress-induced lysosomal membrane permeabilization may release hydrolytic enzymes into the cytoplasm, while a large amount of lipofuscin is accumulated due to the direct effect of heat stress, which triggers excessive autophagy and neuronal death. Overexpression of miR-712-3p likely attenuates this cascade, thereby conferring neuroprotection. The simultaneous accumulation of undegraded autophagic substrates (e.g., lipofuscin), immature autophagic vacuoles, and lipid droplets—as resolved by TEM—constitutes a canonical cytopathological signature of compromised autophagosome-lysosome fusion or lysosomal degradation. Upon miR-712-3p intervention, this pathological morphology was coordinately restored. This synergistic rescue points to the miRNA’s action on a critical regulatory node in the autophagy-lysosome pathway. However, it is not clear how lysosomes are affected by miR-712-3p and through which pathway to inhibit lysosomal activation, which is also the limitation of this study. By querying the miRDB database for genes associated with miR-712-3p, we predicted Atp6v1c1 as a candidate. This gene encodes a key subunit of the vacuolar ATPase complex responsible for H+ transport in lysosomes. Its function is critical in preventing lysosomal hyperacidification and mitigating inflammatory responses. In the future, to directly establish whether lysosomal alkalinization impairs autophagic flux, we will perform mCherry-GFP-LC3 imaging combined with chloroquine blockade. This will determine whether the accumulation of autophagosomes reflects enhanced initiation or failed degradation. Regarding the specific mechanism by which miR-712-3p affects the lysosomal function of neurons, we will employ 3′UTR luciferase assays to verify targeting, corroborate this interaction *via* AGO2-RIP/CLIP, and finally, conduct rescue experiments. In summary, miR-712-3p protects neuronal function and subsequent modulation of lysosomal function of neuron under HS, which astrocyte-derived exosome-enriched preparations also mitigate such injury *in vitro*.

## Data Availability

The data presented in the study are deposited in the GEO repository, accession numbers GSE327014 and GSE327120.
